# Reciprocity between abscisic acid and ethylene at the onset of berry ripening and after harvest

**DOI:** 10.1186/1471-2229-10-257

**Published:** 2010-11-22

**Authors:** Liang Sun, Mei Zhang, Jie Ren, Jianxun Qi, Guojun Zhang, Ping Leng

**Affiliations:** 1 College of Agronomy and Biotechnology, China Agricultural University, Yuanmingyuan West Road, Beijing, PR China

## Abstract

**Background:**

The ripening of grape berry is generally regulated by abscisic acid (ABA), and has no relationship with ethylene function. However, functional interaction and synergism between ABA and ethylene during the beginning of grape berry ripening (véraison) has been found recently.

**Results:**

The expressions of *VvNCED1 *encoding 9-*cis*-epoxycarotenoid dioxygenase (NCED) and *VvGT *encoding ABA glucosyltransferase were all increased rapidly at the stage of véraison and reached the highest level at 9th week after full bloom. However, *VvCYP1 *encoding ABA 8'-hydroxylase and *VvβG1 *encoding berry β-glucosidase are different, whose expression peak appeared at the 10th week after full bloom and in especial *VvβG1 *remained at a high level till harvest. The *VvACO1 *encoding 1-aminocyclopropane-1-carboxylic acid (ACC) oxidase, the *VvETR2 *(ethylene response 2) and *VvCTR1 *(constitutive triple response 1) had a transient expression peak at pre-véraison, while the *VvEIN4 *(ethylene insensitive 4) expression gradually increased from the véraison to one week before harvest stage. The above mentioned changes happened again in the berry after harvest. At one week before véraison, double block treatment with NiCl_2 _plus 1-methylcyclopropene (1-MCP) not only inhibited the release of ethylene and the expression of related genes but also suppressed the transcription of *VvNCED1 *and the synthesis of ABA which all might result in inhibiting the fruit ripening onset. Treatment with ABA could relieve the double block and restore fruit ripening course. However, after harvest, double block treatment with NiCl_2 _plus 1-MCP could not suppress the transcription of *VvNCED1 *and the accumulation of ABA, and also could not inhibit the start of fruit senescence.

**Conclusion:**

The trace endogenous ethylene induces the transcription of *VvNCED1 *and then the generation of ABA followed. Both ethylene and ABA are likely to be important and their interplaying may be required to start the process of berry ripening. When the level of ABA reached the peak value, part of it will be stored in the form of ABA-GE. While after harvest, abiotic stresses principally (such as dehydration, harvest shock) could induce the transcription of *VvNCED1 *and the accumulation of ABA, thus starting the process of fruit senescence.

## Background

Fruits can be classified into two groups, climacteric- and non-climacteric fruit based on its pattern of respiration during maturation and ripening [1]. To date, the researches on the regulatory and signaling pathways of climacteric fruit ripening have been making substantial progress at the molecular level [2-6], especially in tomato, largely through investigation of ripening-deficient mutants, such as *Never-ripe *(*Nr*) [7-9] and *Green ripe *(*Gr*), *Never-ripe2 *(*Nr2*) etc [10]. The tomato mutants highlighted this aspect, that is, ethylene is important for many aspects of fruit ripening [11-13] but other factors, such as the MADS box transcription factors, are higher in the hierarchy of development regulation, and act also independently of the ethylene pathway [11,14]. In addition, both ethylene-dependent and independent regulation of ripening pathways have been found in climacteric fruit, such as tomato and melon [15-17]. Compared with climacteric fruits, the information we have about the molecular regulation mechanisms of non-climacteric fruit ripening is notably less. Generally, as a non-climacteric fruit, the ripening of grape is regulated by ABA and the role of ethylene is usually negligible. Roles of ABA in plants and genes involved in ABA catabolism and biosynthesis have been extensively studied in recent years [18,19]. NCED is known as the key enzyme in the biosynthesis pathway of ABA [20-22]. However, the expression of *NCED *gene is always not consistent with the level of ABA, conjecturing that more complex regulation mechanism lies in the accumulation of ABA. In fact, endogenous ABA content is modulated by a dynamic balance between biosynthesis and catabolism, which are regulated by *NCEDs *and *CYP707As *transcripts, respectively, in tomato [18,23,24] and cherry fruits [25]. ABA can be either degraded, through the irreversible pathway starting with 8' hydroxilation, catalyzed by ABA 8'-hydroxylase (*CYP707As*), or stored in a bound form ABA-glucosylester (ABA-GE), catalyzed by ABA glucosyltransferase (ABA-GTase) [26-28]. The glucosyltransferase (GTase) is thought to play an important role in the biosynthesis of many plant secondary metabolites. It can transfer nucleoside diphosphate-activated sugars to receptors of low molecular weight substrates. ABA-glucosyl ester (-GE) could be synthesized from ABA and UDP-D-Glc (UDPG) by a GTase [29-31]. ABA-GE is one of the ABA metabolite and plays an important role in the regulation of ABA level [31]. Early observations suggested that ABA-GE is an inactive end product of ABA metabolism [27]. Recently, it was reported [19] that the *Arabidopsis thaliana *β-glucosidase1 (AtβG1) catalyzed the release of ABA-GE back into active ABA to rapidly adjust ABA level. Above contents suggest that the key steps of ABA hormonal homeostasis in higher plants responding to plant developmental and stressful cues is regulated either by NCED and CYP in the pathway of synthesis-degradation or by GT and βG1 in the pathway of conjugation-dissociation. In addition, level of ABA in plants is susceptible to the effect of environmental stress, such as salting, low temperature and water deficiency, resulting in sharp increase of the ABA level. Environmental stresses can not only induce the synthesis of ABA, resulting in increase its level, but also can promote the metabolism of ABA, producing PA, DPA and so on to decrease its level. Therefore, both ABA synthesis and metabolic pathways and environmental stresses have different degrees of effect on the level of ABA in plants [32]. Further researches are needed to clarify their contribution ratio respectively.

Recently, it was pointed out that in some non-climacteric fruits there was indeed a small amount of ethylene been synthesized and transient expression of ethylene responsive genes. For examples, citrus fruits displayed property of autocatalytic in ethylene synthesis [33]; exogenous ethylene could regulate the expression of ethylene receptor-related genes *ETR1 *and *ERS1 *(ethylene response sensor 1) in grape [34,35]; ethylene peak was detected at onset of ripening in grape and strawberry [36,37]; and the expression of some ripening-related genes was found to be up-regulated in strawberry [38] etc. In addition, application of exogenous ethylene at the beginning of grape berry ripening could induce changes in ripening progress [39], accelerate the development process of grape berry, make fruit bigger, increase the accumulation of anthocyanin [36] and change the transcription levels of many genes [37]. The results described above indicate that ethylene plays a role in the ripening process of non-climacteric fruit, such as grape. So it is necessary to do some further studies on the effects of ethylene on the ripening of non-climacteric fruits.

Grape (non-climacteric fruit) and tomato (climacteric fruit) display the same ripening phenomenon in terms of the expression of genes related with cell wall degradation, pigment synthesis and sugar synthesis increases gradually and the activity of the corresponding enzymes increases continually, with only a difference in ethylene production. We suggest that grape berry may have the similar ripening regulation pathway as tomato, a climacteric fruit. ABA may be the common ripening regulation factor for both two types of fruit, as ABA increased rapidly after the beginning of fruit ripening [40] and had high correlation with accumulation of sugar, reduction of acid [41,42] and production of fruit pigments [43-45,33].

It has been found recently that ABA could stimulate the autocatalysis of system II ethylene by regulating the expression of *ACS *and *ACO*, thus starting the ripening process of tomato, while ethylene plays an important role in the later stage of fruit ripening, especially in the process of intrinsic flavor forming [46-48]. This result suggested a functional sympathetic and interactions between ABA and ethylene. However, how was induced for the transcription of NCED at the beginning of fruit ripening? What are the respective roles of ethylene and ABA during the process of grape berry ripening? Is there a functional sympathetic interaction between them? In order to answer those questions, we carried out this experiment.

In this study, we firstly investigate the changes in ethylene, ABA and the related regulation factors in the process of grape berry ripening and then verify through pharmacological experiments the effects of endogenous ethylene and exogenous ABA on the start of grape berry ripening (véraison) and the beginning of post-harvest berry senescence.

## Results

### Development of grape berries and the changes of ABA and Ethylene

**Figure 1:**
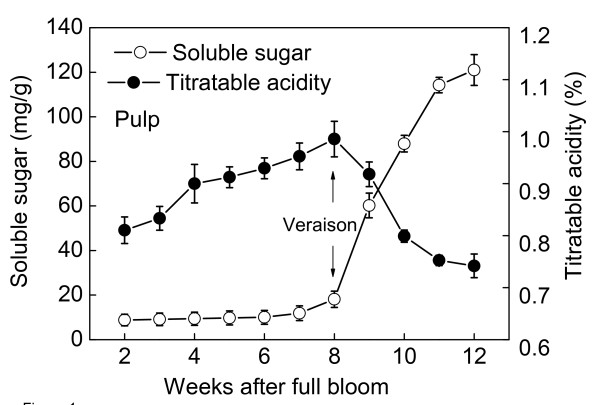
**Changes of soluble sugar content and titratable acidity in berries of Muscat Hamburg during grape development**. The experiments were repeated 3 times, and the bars represent SE.

**Figure 2:**
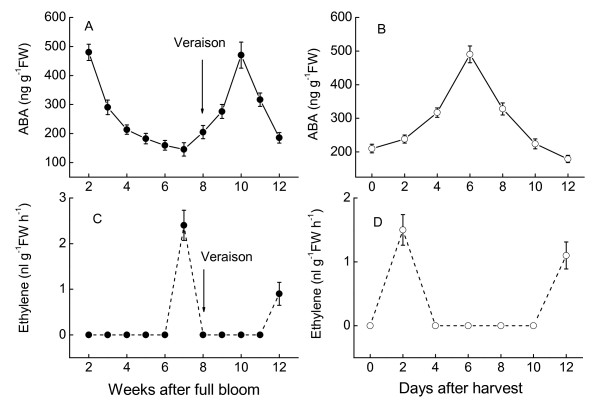
**Changes of ABA and ethylene content in berries of Muscat Hamburg during grape development**. The experiments were repeated 3 times, and the bars represent SE.

The dynamic processes of Muscat Hamburg grape berry development are divided into three major phases during a period of 12 weeks after full bloom [49]:1-3rd weeks for the first rapid berry growth phase (Phase I), 4-8th weeks for the lag phase (Phase II) and 9-12th weeks for the second rapid berry growth phase (Phase III). Morphologically the berry color changes from dark green to light green and further to red green at 8-10th weeks after full bloom (WAFB). As shown in Figure 1, soluble sugar content (mainly glucose and fructose) began to increase rapidly at the 8th WAFB while acid content began to decrease sharply, which mean the onset of grape berry ripening (véraison). The accumulation of ABA decreased during the first growth phase until the immature green stage. At véraison, the level of ABA increased promptly and transiently reaching its maximum at 10 WAFB (Figure 2). The content of ABA in harvested berries reached a maximum at the 6th days after harvest and then quickly decreased. On the other hand, we examined the release of ethylene at the 7th WAFB, one week before the ABA increase. After harvest, a peak of ethylene was observed on the second day. The peak value of ethylene was very low (Figure 2), but consistent with earlier reports [36,50].

### Expression of *VvNCED1*, *VvCYP1*, *VvGT *and *VvβG1 *genes during grape berry growth and ripening

**Figure 3:**
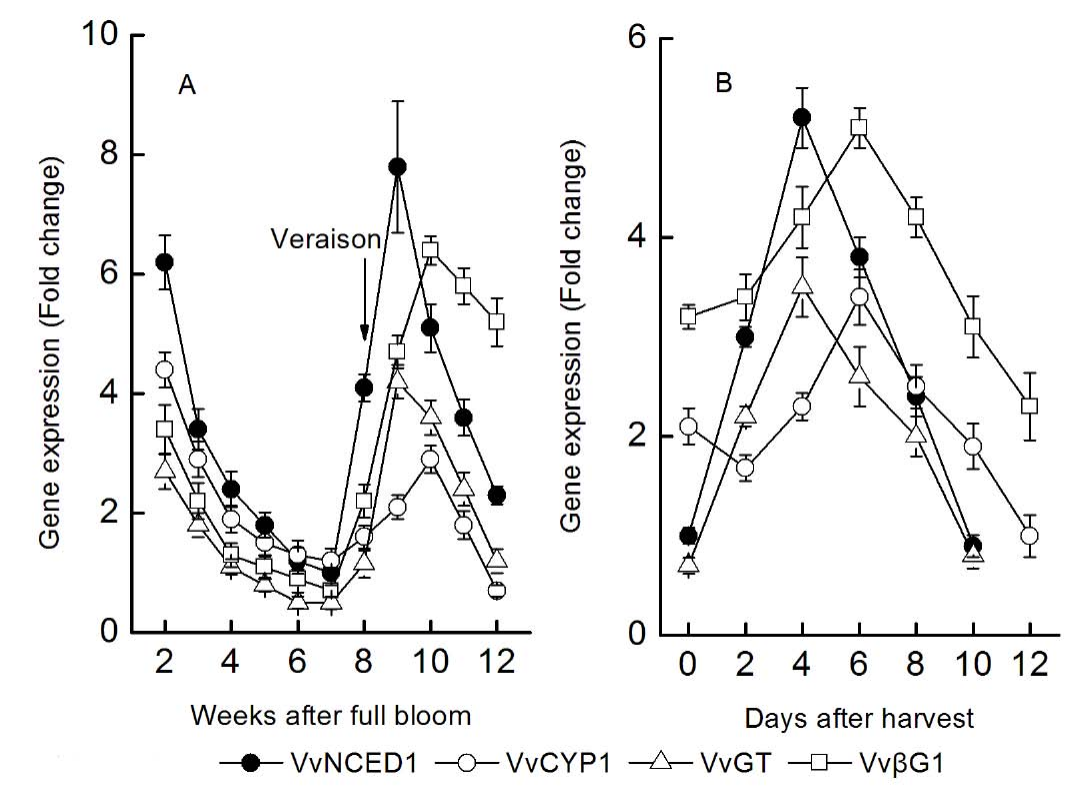
**The expression of *VvNCED1, VvCYP1, VvGT *and *VvβG1 *genes during grape berry growth and ripening of Muscat Hamburg (*Vitis vinifera *L**.). Real time RT-PCR was performed on total RNA isolated from pulp collected in the 2007 seasons. *VvACT *was used to standardize each reaction run with respect to RNA integrity, sample loading, and inter-PCR variations. Expression of each gene is presented as relative fold change. Values presented are means ± SE (n ≥ 3).

The expression patterns of these genes during grape berry development are shown in Figure 3. The *VvNCED1 *gene was expressed highly at the second WAFB, and then decreased gradually with the berry development. It was increased again from onset of ripening and reached the peak value at the 9th WAFB. This trend is almost consistent with the accumulation of ABA. The expression pattern of *VvGT *genes is almost the same as *VvNCED1 *but the expression level is lower. Compared with these two genes, *VvCYP1 *and *VvβG1 *had similar change at early stage, but their expression peak occurred later at the 10th WAFB and *VvβG1 *only remained at a high level till fruit harvest. After harvest, the expression of *VvNCED1, VvCYP1, VvGT *and *VvβG1 *arrived at the peak value on the 4th or 6th day and then declined to the lowest point. Berry also took on a stage of senescence.

### Expression of *VvACO1*, *VvETR2, VvCTR1 *and *VvEIN4 *genes during grape berry development

**Figure 4:**
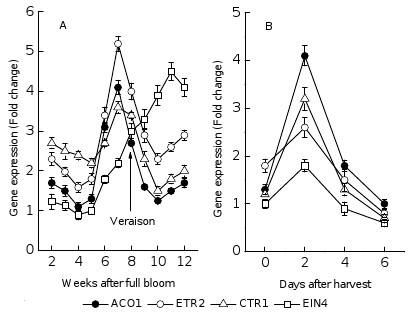
**The expression of *VvACO1, VvETR2*, *VvCTR1 *and *VvEIN4 *during grape berry development of Muscat Hamburg (*Vitis vinifera *L**.). Real-time RT-PCR was performed on total RNA isolated from pulp, and *VvACT *was used to standardize each reaction run with respect to RNA integrity, and inter-PCR variations. Expression of each gene is presented as relative fold change. The experiments were repeated 3 times, and the bars represent SE (n ≥ 3).

Figure 4 shows the expression of *VvACO1 *encoding the key enzyme of ethylene synthesis and the signal transduction-related genes *VvETR2, VvCTR1 *and *VvEIN4*. The expression of *VvACO1, VvETR2 *(ethylene response 2) *and VvCTR1 *(constitutive triple response 1) had an obvious peak value at the 7th WAFB (one week before véraison) which was consistent with the ethylene peak. The expression of *VvEIN4 *(ethylene insensitive 4) increased from the beginning of ripening till the week before harvest. After harvest, these four genes reached a peak value on the second day and then rapidly reduced to low levels.

### Effects of NiCl_2 _plus 1-MCP and ABA treatment on the beginning of grape berry ripening

**Figure 5:**
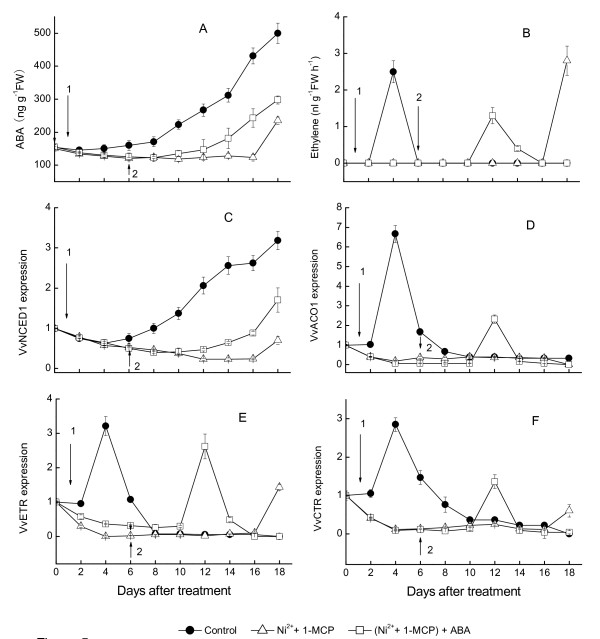
**Effects of double treatment with NiCl_2 _plus 1-MCP, and treatment with ABA on the beginning of grape berry ripening of Muscat Hamburg (*Vitis vinifera *L**.). Expression of each gene is presented as relative fold change. The three replicates were conducted for each treatment, and the bars represent SE (n = 3). The arrow-1 (↓) indicates the treatment with NiCl_2 _plus 1-MCP, and arrow-2 (↓) indicates the treatment with ABA 5 days after with NiCl_2 _plus 1-MCP treatment.

In order to investigate the role of trace endogenous ethylene in the beginning of ripening, experimental clusters on the vine in field were selected at the 7th WAFB and divided into three groups for following treatments. First and second group of grape clusters were treated with both 0.1% (w/v) of Ni^2+ ^for 10 min and 5 μl L^-1 ^of 1-MCP for 24 h to block ethylene. To evaluate the role of ABA, the second group of clusters was again treated 5 d later after both Ni^2+ ^and 1-MCP treatment with an ABA solution of 100 μM. The third group was control (no treatment). Ethylene could be detected at 4 d after treatment; however, ABA increased gradually since one week after véraison (Figure 5A). Meanwhile, *VvNCED1 *expression increased at 6 d after treatment, reaching a maximum at 18 d, and *VvACO1*, *VvETR2 *and *VvCTR1 *expression increased at 4 d after treatment, was consistent with ethylene in the control berry. Compared with the control, Ni^2+^-and 1-MCP treatment effectively inhibited ABA content, ethylene production and the expression of *VvNCED1*, *VvACO1*, *VvETR2 *and *VvCTR1 *(Figure 5). The berries treated with both Ni^2+^-and 1-MCP remained in ripening onset stage, while the control fruit had gradually ripened further after véraison. At 6th days after ABA treatment, the expression of *VvNCED1*, *VvACO1*, *VvETR2 *and *VvCTR1*, as well as ethylene synthesis were restored and increased, then berries turning light green. The fruits treated only by both Ni^2+ ^and 1-MCP were dark green until 18th days after treatment. As berries treated by both Ni^2+^and 1-MCP did not develop a light green, even beyond 18 d, no ripening-associated ethylene production was expected.

### Effects of NiCl_2 _plus 1-MCP and ABA treatment on starting of grape senescence after harvest

**Figure 6:**
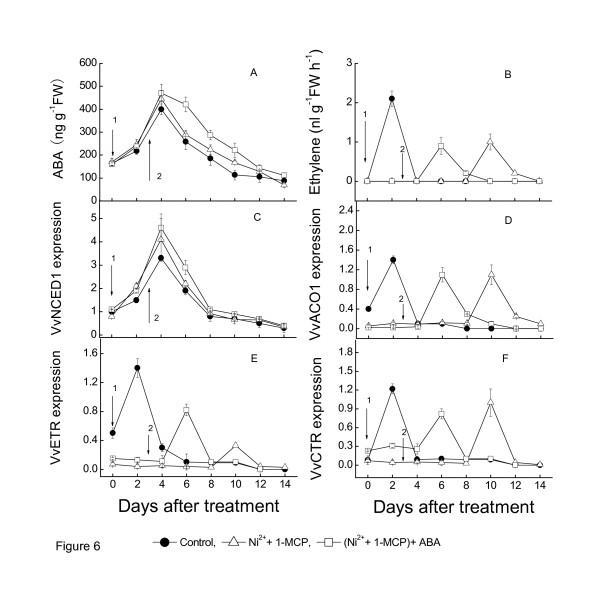
**Effects of double treatment with NiCl_2 _plus 1-MCP, and treatment with ABA on starting of grape senescence after harvest**. Expression of each gene is presented as relative fold change. The three replicates were conducted for each treatment, and the bars represent SE (n = 3). The arrow-1 (↓) indicates the treatment with NiCl_2 _plus 1-MCP, and arrow-2 (↓) indicates the treatment with ABA 3 days after with NiCl_2 _plus 1-MCP treatment.

In order to investigate the effects in both Ni^2+ ^plus 1-MCP treatments and ABA treatment on fruit senescence after harvest, the grape clusters were harvested at the 12 WAFB, divided into three groups and treated at first with both NiCl_2 _+1-MCP and 3 d later with ABA. The result is shown in Figure 6. Double treatment with NiCl_2 _and 1-MCP could not effectively suppressed the transcription of key enzyme gene *VvACO1 *in ethylene synthesis and ethylene receptor-related genes *VvETR2*, *VvCTR1 *and *VvEIN4*, which showed only some delay and weakness. On the other hand, double treatment could neither inhibit the transcription of *VvNCED1 *and ABA content, nor stimulated their production. Double treatment with NiCl_2 _and 1-MCP could not delay the start of grape senescence.

### Effects of exogenous ABA and ethephon treatments on the expression of *VvNCED1 *and *VvACO1*, and the contents of ABA and ethylene

**Figure 7:**
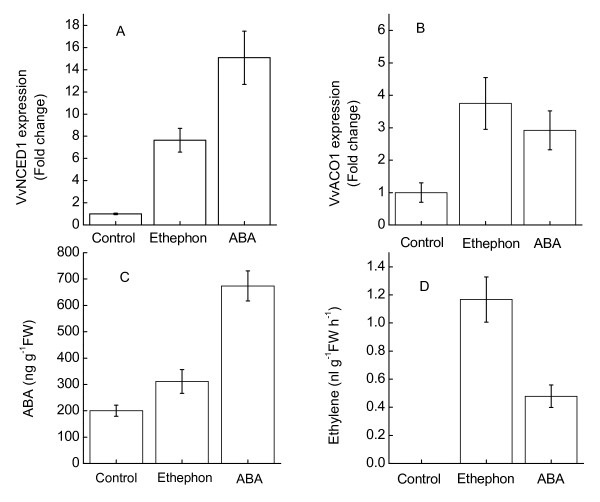
**Effects of exogenous ABA and ethephon treatments on the expressions of *VvNCED1 *and *VvACO1*, and the contents of ABA and ethylene**. Expression of each gene is presented as relative fold change. Data are the means ± standard deviation of three measurements (n = 3).

Three days after treatment, there was a significant increase in ABA levels in the ABA-treated pulp compared with the levels in the control pulp samples (Figure 7C). There was a slight but statistically significant increase in the ABA level in the ethephon treated pulp. Real-Time PCR analysis revealed that *VvNCED1 *transcript levels were much higher in pulp treated with ABA and ethephon (Figure 7A).

Ethephon and ABA treatment could stimulate the expression of *VvACO1 *(Figure 7B). In agreement with this, the ethylene evolution in grape berry was induced by ethephon and ABA treatment (Figure 7D). These results indicated that the ethephon and ABA treatments could stimulate ethylene evolution and ABA accumulation via the transcripional regulation of the respective biosynthesis genes.

### Changes in ABA content and quantitative expression analysis during berries development

**Figure 8:**
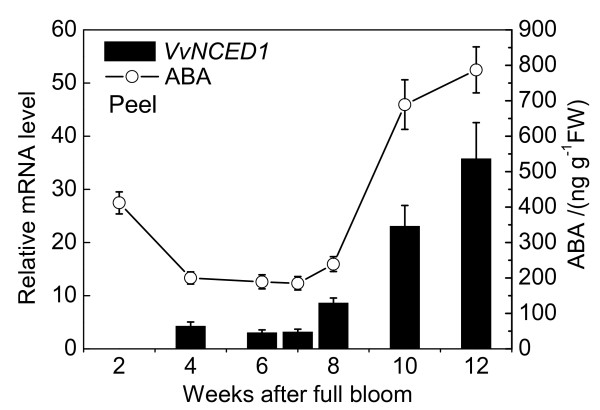
**Changes in ABA content and quantitative expression analysis of berries during development**. Real time RT-PCR was performed on total RNA isolated from peel. *Vvactin *was used to standardize each reaction run with respect to RNA integrity, sample loading and inter-PCR variation. Expression of each gene is presented as relative fold change. Data are the means ± standard deviation of three measurements (n = 3).

**Figure 9:**
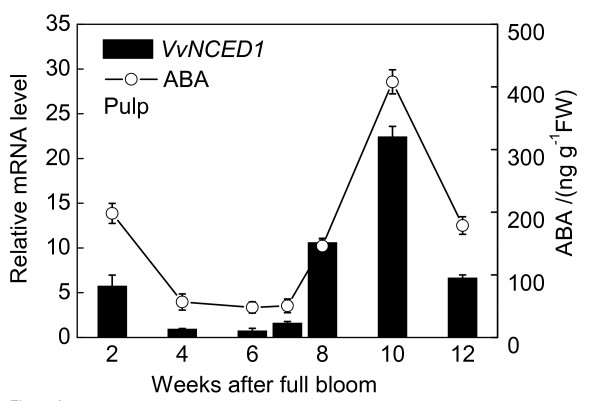
**Changes in ABA content and quantitative expression analysis of berries during development**. Real time RT-PCR was performed on total RNA isolated from pulp. *Vvactin *was used to standardize each reaction run with respect to RNA integrity, sample loading and inter-PCR variation. Expression of each gene is presented as relative fold change. Data are the means ± standard deviation of three measurements (n = 3).

**Figure 10:**
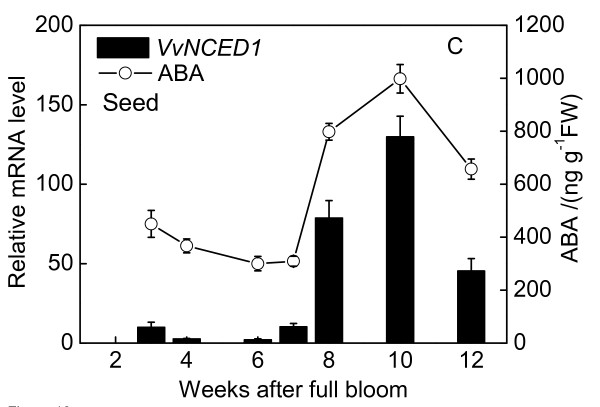
**Changes in ABA content and quantitative expression analysis of berries during development**. Real time RT-PCR was performed on total RNA isolated from seed. *Vvactin *was used to standardize each reaction run with respect to RNA integrity, sample loading and inter-PCR variation. Expression of each gene is presented as relative fold change. Data are the means ± standard deviation of three measurements (n = 3).

As Figures 8, 9, 10 shown, tissue-specific changes in endogenous ABA content during fruit development were determined. The tissues analyzed included pulp, peel and seed. Real-Time RT-PCR was performed with RNA isolated from pulp, peel and seed to analyze relative fold of *VvNCED1 *gene expression during fruit maturation.

The ABA level of pulp in 2007 season at 2 WAFB was relatively high, but fell to lower level at 6-7 WAFB, and then the ABA content began to increase after 8 WAFB when the first rapid growth period ended, coincident with an increase in skin color due to anthocyanin accumulation, the accumulation of sugars and decrease in acidity and firmness. After peaking at 10 WAFB, the level of ABA declined thereafter. The ABA level in seed followed a similar pattern to that in the pulp. The ABA content of peel increased dramatically after 10 WAFB and reached the highest level at 12 WAFB. The transcript levels of *VvNCED1 *in pulp, seed and peel tissues were measured throughout development by Real-Time PCR analysis. The expression of *VvNCED1 *was coincident with the timing and pattern of ABA accumulation.

## Discussion

### Effects of ABA and ethylene on ripening onset of grape berry

Our data shows that there are three ABA peaks during the process of grape berry growth, ripening and post-harvest senescence. The first time appeared at the immature stage shortly after full bloom which may be related with the division of pulp cells. The second peak was at about 10 WAFB and it may have relationship with the regulation of grape berry ripening. The third is appeared at the sixth day after harvest and it resulted from fruit senescence. It may be conjecturable that grape berry got senescence rapidly when ABA reaching its peak value after harvest, and the process is irreversible in the grape berry. *VvNCED1 *is the key enzyme gene in ABA biosynthesis and the very ABA exists in the whole process of fruit growth, ripening and senescence. ABA is an important regulator of grape ripening. Apart from the regulation of *VvNCED1 *in grape berry, the transcription of ABA degradation enzyme gene *VvCYP1*, glucoxyltransferase gene *VvGT *and glucosidase gene *VvβG1 *also affect ABA level. Figure 3 shows that the expression of *VvβG1 *remained at a high level from coloration to fruit ripening, which indicates that ABA produced by *VvβG1 *plays an important role in regulating the level of ABA during the later stage of ripening [19] and it may be more important than *VvNCED1*. Moreover, the accumulation of ABA in fruit also has correlation with abiotic stresses [51-53]. Part of ABA may store in the form of ABA-GE by the time it reached the peak value at the 10th WAFB. When the grape berry is subjected to abiotic stresses or physiological disorders, ABA releases from the ABA-GE form catalyzing by *VvβG1 *and exerts physical function. *VvGT *mainly relates with the down-regulation of ABA and plays a role in regulating the level of ABA around fruit véraison. The expression of *VvCYP1 *is higher at the immature stage which has the same variation trend with *VvNCED1*, indicating that the level of ABA is regulated by both its synthase key enzyme gene and degradation enzyme gene during the immature stage and around véraison. The transcription level of *VvCYP1 *is low during fruit ripening, indicating that it has a small effect on regulation of ABA at ripening stage. *VvNCED1 *is the key enzyme gene in ABA biosynthesis, but what is the inducing factor for the increased transcription of *VvNCED1*? Based on the expression pattern of *VvNCED1 *and *VvACS *in the grape fruit development, Deluc [52] concluded that ethylene may involve in the induction of ABA biosynthesis, but there is no further evidence. Recently, the problem about functional interactions between ethylene and ABA signal has been paid great attentions. In tomato [46], the accumulation of ABA is earlier than the synthesis of ethylene and treating tomato fruit with ABA can induce the expression of ACC synthase genes and ACC oxidase genes, indicating that there is an interaction between this two signal pathways.

Grape is non-climacteric fruit; therefore, for a long time people think there is little correlation between grape ripening and ethylene. However, recent study shows that ethylene may play an important role in grape ripening. We detected the transcription peak of *VvACO1 *, *VvETR2 *and *VvCTR1 *just before véraison (at the 7th WAFB) and this time was consistent with the appearance of ethylene peak. This result indicates that both ethylene synthesis and signal transduction pathway are active around grape berry véraison, so we suppose that they are related with the start of fruit ripening. Similar expression patterns have been found at the ripening onset of strawberry [38] and at mature green stage of tomato [54]. According to the early report [54], treatment with exogenous ethylene could increase the transcription level of ethylene receptor and reduce the level of corresponding proteins. All these results suggest ethylene may play an important role in triggering the start of grape ripening.

### Reciprocity between ABA and ethylene at the onset of berry ripening and after harvest

The trace ethylene is active when expression of *VvNCED1 *increases gradually in the véraison [55], guessed that trace endogenous ethylene may induce the expression of *VvNCED1 *and also the accumulation of ABA. In order to verify this conjecture, we treated grape clusters one week before véraison with both NiCl_2 _and 1-MCP to double block ethylene. 1-MCP could bind to ethylene receptors which led to the inhibition of ethylene signal, while NiCl_2 _could suppress the activity of ACC oxidase in fruits. Therefore, treating fruits with NiCl_2 _and 1-MCP at the same time could block ethylene signal at two different points, namely upstream and downstream of the ethylene signal pathway. The results showed that double blocking treatment before véraison of grape berry not only inhibit the production of ethylene effectively but also suppress the transcription of *VvNCED1 *and the production of ABA. The results indicate that trace endogenous ethylene induces the transcription of *VvNCED1 *and then the accumulation of ABA follows at the beginning of berry ripening. However the signal source of trace ethylene is unclear at present. Contrarily, double treatment with NiCl_2 _and 1-MCP after harvest stimulated the transcription of *VvNCED1 *and the increase of ABA (Figure 6A and 6C). Grape berries will suffer a certain degree of water stress for losing the supply of water from parent plant after harvest. Moreover, the grape berries will also suffer a certain degree of non-biological damages in the harvesting process, such as injury, extrusion, collision and so on. We suppose that these abiotic factors induced the transcription of *VvNCED1 *and the increase of ABA after harvest. The increase of ABA to a certain level may start berry senescence. ABA inhibits the ethylene synthesis in young stage (dark green berries) and stimulates ethylene release in the véraison (light green berries) (data not shown). These data suggest that ABA exerts different actions in ethylene biosynthesis at different stages of grape development, and ABA seems to regulate the transition to autocatalytic ethylene production via signal transduction, an expression of ethylene biosynthetic genes. When grape berries were harvested and kept in the laboratory at room temperature and humidity (40 to 50% RH), ABA levels increased with the water loss of pedicle. Ripening ethylene was subsequently produced, and then the berries separated from pedicle, followed by berries over ripening. On the other hand, when the pedicle was supplied with sufficient water, ABA content in berries gradually declined, ripening ethylene was not produced, the berries did not separate from the pedicle, and the flesh did not become soft during the experiment (data not shown). Moreover, because of the continuous metabolism, the whole berries gradually lightened, shrank, metamorphosed, but did not become soft. Thus, ABA appears to trigger the onset of senescence in detached grape berries after harvest. Before endogenous ABA reached the peak level, exogenous application of ABA could induce ethylene and accelerate berry ripening. However, this effect was gradually getting weaker during the progress of berries ripening. After endogenous ABA reached the peak level, exogenous ABA had no effect on ethylene synthesis, although ethylene treatment could still accelerate berries softening. Thus, the role of ABA in triggering grape berries ripening only depended on the ripening stage, not the other aspects such as varieties or growth conditions. The data we obtained from the experiment on grape berry development may have significance for further discovering the mechanism of ABA-dependent regulation of fruit ripening for grape and other non-climacteric fruits. In the other hand, ABA content of seeds is several times higher than that of pulp tissue [56], therefore, it was regarded generally that ABA in seeds was the ABA source for fruit ripening. However we found that the ABA content in pulp around the seeds was much lower and it didn't decreased with the distance from seeds, which indicated that ABA was independently synthesized both in pulp and seed during fruit development (Figure 9 and 10). ABA produced by pulp or seeds regulated fruit ripening and seed maturity, respectively. Recently, novel candidates have been identified for regulating nonclimacteric fruit ripening, and it was demonstrated that grape orthologs of key sugar and ABA-signaling components are regulated by sugar and ABA in grape berries [57].

## Conclusions

We propose that for grape berries to begin ripening, trace endogenous ethylene may induce the expression of *VvNCED1 *to encode a key enzyme in ABA biosynthetic pathway, and then generation of ABA follows. The accumulation of ABA to a certain level can induce the expression of ABA-dependent ripening-related genes by signal transduction system [58], cause berries ripening. After this, part of ABA may be stored as ABA-GE form. When berries were subjected to stress by harvest shock, ABA was increased again after harvest and accumulation of ABA to a higher level can start process of berry senescence.

## Methods

### Berry sampling

Twelve-year-old grapevines (*Vitis vinifera *L. cv. Muscat Hamburg) are grown in the vineyard on the campus of China Agricultural University, Beijing, China. The experimentation has been performed in 2007, full bloom of grapevines occurred in mid-May and the onset of grape berry ripening (véraison) occurred on 8th week after full bloom (WAFB). Samples were taken once a week from the second WAFB and immediately frozen in liquid nitrogen and stored at -80°C until required. To define the stage of berry development, 30 randomly selected berries were weighed and an average berry weight was calculated. The level of total soluble sugar and titratable acidity were measured.

Tissue-specific changes in endogenous ABA content during fruit maturation were determined. The tissues analyzed included pulp, peel and seed. Real-Time RT-PCR was performed with RNA isolated from pulp, peel and seed to analyze relative fold expression of *VvNCED1 *during fruit maturation.

### Drugs used in this experiment

Abscisic acid (ABA): (±)-Abscisic acid, ≥ 98.5% (HPLC); CAS Number: 14375-45-2; Sigma, Product Number: A1049.

Nordihydroguaiaretic acid (NDGA): purum, ≥ 97.0% (HPLC) CAS Number: 500-38-9; Sigma, Product Number: 74540.

### Effects of NiCl_2 _plus 1-MCP and ABA treatment on ethylene and *VvNCED1 *expression

In order to investigate the role of trace endogenous ethylene in the beginning of ripening, experimental clusters on the grape plants were selected at the 7th WAFB (one week before véraison) and divided into three groups for following treatments. First and second groups of grape clusters were treated with both Ni^2+^- and 1-MCP to block ethylene by direct soaking the clusters into NiCl_2 _solution (0.1% (w/v)) for 10 min (using plastic cups), the clusters after being dried were bagged and sealed in polyethylene bags into which were injected 5 μl L^-1 ^of 1-MCP for 24 h according to Nakatsuka et al. [59]. To evaluate the role of ABA, the second group of clusters was again treated 5 days later after both Ni^2+^- and 1-MCP treatment with an ABA solution of 100 μM using the same method described above. The remaining third group of clusters was used as a control without any treatment. The measurements of ethylene, ABA and *VvNCED1 *mRNA were initiated 2 days after the completion of the Ni^2+ ^and 1-MCP treatment, and three replicates (three clusters for each replicate) were conducted for each treatment and ten berries from each cluster were chosen for analysis and determination.

In order to investigate the effects in both nickel ion and 1-MCP treatments and ABA treatment on fruit senescence after harvest, the grape clusters were harvested at the 13th WAFB, divided into 3 groups and treated at first with both NiCl_2 _plus 1-MCP and 3 days later with ABA. The treating methods, sampling and detections were the same as described above.

### Effects of exogenous ABA and ethephon treatments

In order to evaluate the effect of exogenous ABA and ethephon treatment, grape berries were treated at 6th week after full bloom (2007-7-1) on the grapevine. They were divided into 3 groups and used for the following treatments: ABA (100 μM, group 1), ethephon (500 μM, group 2), and control (distilled water, group 3). The berries were treated by dipping in the solutions or distilled water, respectively. Three replications were conducted for each solution, with each treatment containing 3 cluster berries. Berries were sampled at 3 days after treatment (2007-7-3) for measurement of the ABA content and ethylene.

### RNA Extraction, Reverse Transcription-PCR (RT-PCR), and Sequencing

Total RNA was extracted from 1 g of flesh using the hot borate method [60]. Synthesis of the first-strand cDNA from 2 μg of total RNA was conducted using a Moloney murine leukemia virus reverse transcriptase (Takara, Dalian, PR China). The cDNA was used as a template for amplifying *NCEDs *with degenerate primers (forward, 5'-TTYGAYGGIGAYGGIATGGTICA-3'; reverse, 5'-TCCCAIGCRTTCCAIARRTGRAA-3') designed from the conserved sequences of plant *NCEDs *(AF224671, Z97215, DQ028471, DQ028472, AY337613). To obtain the 3' nucleotide sequences, RACE-PCR was performed using the Kit (5'/3'RACE System for Rapid Amplification of cDNA Ends, invitrogen™) according to the manufacturer's instructions. The PCR products were sequenced by Invitrogen (Shanghai, PR China).

### Real-time quantitative PCR

First-strand cDNA synthesis was conducted using the PrimeScript™RT reagent kit (TaKaRa) from 1.0 μg total RNA. The sequences of the primer pairs used for each gene are shown in Table 1. Real-time PCR was performed using the SYBR Premix Ex Taq™kit (TaKaRa). Reactions contained 1 μl of primer mix, 2 μl cDNA template, 10 μl SYBR Premix Ex Taq™(2×) mix and 7 μl water for a total volume of 20 μl. Reactions were carried out under the following conditions: 95°C/30 s (1 cycle); 95°C/15 s, 58°C/20 s; 72°C/15 s (40 cycles), using a Rotor-Gene 3000 system (Corbett Research, Australia). The PCR product of each gene was confirmed by agarose gel electrophoresis and double-strand sequencing. The amplified fragment of each gene was subcloned and used to generate efficiency curves. Relative fold expression for each gene was calculated by the Rotor-Gene 6.1.81 software using "two standard curves method" in which the value of young fruit stage (or lower value sample) was set at "1". The transcript of *actin *gene was used to standardize each reaction run with respect to RNA integrity, sample loading and inter-PCR variations.

**Table 1:** Specific primers used for amplification of genes.

Name	Oligonucleotides	GenBank number
VvNCED1-F	5'-GGTGGTGAGCCTCTGTTCCT-3'	AY337613
VvNCED1-R	5'-CTGTAAATTCGTGGCGTTCACT-3'	
VvCYP1-F	5'-GGTCACTTGGAGGGTAATTAC-3'	XM_002282197
VvCYP1-R	5'-TGTTGTCGGCGATTTGATCCT-3'	
VvGT-F	5'-GGGTCGGTTGTCAACAG-3'	XM_002285067
VvGT-R	5'-TCCTCCGATGGCGGCGTGTTC-3'	
VvβG1-F	5'-GGTGACAGGGTGAAGCACTG-3'	XM_002274626
VvβG1-R	5'-GGCAACCGGCTTCCTACTCCATC-3'	
VvACO1-F	5'-AAATCCCAGACCTTGAAGAA-3'	AY211549
VvACO1-R	5'-GCCTGGAACTTCAAACCGGC-3'	
VvETR2-F	5'-TTTGCACCAAAAGCATGGCTC-3'	CAN84042
VvETR2-R	5'-GGTTCAGAAATGTTGATTCC-3'	
VvCTR1-F	5'-TGCACAAACCTGGTGCAAGAG-3'	CAO15968
VvCTR1-R	5'-TCATGCCCTTGGCCACA-3'	
VvEIN4-F	5'-GAAGTAGCAAAAAGAATCCG-3'	AM30288
VvEIN4-R	5'-GCTTGCTGTCAGGGCTATG-3'	
Actin-F	5'-GTGCCTGCCATGTATGTTGCC-3'	AF369524
Actin-R	5'-GGTCACGTCCAGCAAGGTCAAG-3'	

### Determination of ABA Content

For ABA extraction, 1 g of flesh was ground in a mortar and homogenized in extraction solution (80% methanol, v/v). Extracts were centrifuged at 10,000 g for 20 min. The supernatant liquid was eluted through a Sep-Pak C_18 _cartridge (Waters, Milford, MA, USA) to remove polar compounds, and then stored at -20°C for enzyme-linked immunosorbent assay (ELISA). The ELISA procedures were conducted according to the instructions provided by the manufacturer (China Agricultural University, Beijing, PR China). ABA was determined by Thermo Electron (labsystems) Multiskan MK3 (PIONEER Co., PR China).

### Determination of Ethylene Production

Ethylene production from the fruit was measured by enclosing three berries in 50 mL centrifuge tube, or one grape cluster in 1.5 L airtight jars for 2 h at 20°C, withdrawing 1 ml of the headspace gas, and injecting it into a gas chromatograph (model Agilent, 6890N, USA) fitted with a flame ionization detector and an activated alumina column. Flesh tissues from each fruit were frozen in liquid nitrogen and stored at -80°C until use.

### Determination of soluble sugar content and titratable acidity

Grape flesh samples (0.5 g) after certain procedures of preparation were used for determination of the soluble sugar content by HPLC **(**Agilent 1200 HPLC, USA), and the titratable acidity was determined by repeated titration with 0.1 mol/L NaOH (2 drops of 1% phenolphthalein added) to a faint pink. Based on the volume of NaOH solution used for titration calculate the titratable acidity expressed as % of tartaric acid content (as grams of tartaric acid per 100 ml of juice).

## Authors' contributions

LS carried out the mRNA extraction, qRT-PCR, medicament treatment, prepared figure and tables and drafted the manuscript. MZ performed all metabolite analyses including HPLC analyses. JR acquired physiological data. JQ and GZ performed the sugar and acid determinations. PL conceived and organized the study, assisted in the ABA analyses, and prepared the final manuscript. All authors read and approved the final manuscript.
